# Porcine Rotavirus Closely Related to Novel Group of Human Rotaviruses

**DOI:** 10.3201/eid1708.101466

**Published:** 2011-08

**Authors:** Mitsutaka Wakuda, Tomihiko Ide, Jun Sasaki, Satoshi Komoto, Junichi Ishii, Takeshi Sanekata, Koki Taniguchi

**Affiliations:** Author affiliations: Fujita Health University School of Medicine, Toyoake, Japan (M. Wakuda. T. Ide, J. Sasaki, S. Komoto, J. Ishii, K. Taniguchi);; Tottori University, Tottori, Japan (T. Sanekata)

**Keywords:** pigs, rotavirus, viruses, phylogeny, sequences, Japan, viral protein, nonstructural protein, dispatch

## Abstract

We determined nucleotide sequences and inferred amino acid sequences of viral protein (VP) 4, VP6, VP7, and nonstructural protein 4 genes of a porcine rotavirus strain (SKA-1) from Japan. The strain was closely related to a novel group of human rotavirus strains (B219 and J19).

Rotaviruses, a member of family *Reoviridae*, are a major etiologic agent of acute gastroenteritis in humans and animals worldwide. Rotaviruses are classified into 7 groups designated A–G (*1**–**3*). Group A rotaviruses cause severe diarrhea in infants and children, and is estimated to be associated with 527,000 childhood deaths annually. They are also responsible for diarrhea in young mammals and birds of various species. Group B rotaviruses were first detected in a large water-borne outbreak of diarrhea among adults in the People’s Republic of China, and were recently found in Bangladesh, India, and Myanmar. They have also been detected in cows, pigs, and rats. Group C rotaviruses cause sporadic and epidemic gastroenteritis in children and adults. They have also been detected in pigs, cows, and other animals. Group E rotaviruses were detected in pigs, and group D, F, and G rotaviruses were detected in chickens.

The complete nucleotide sequence of the genome of an avian group D rotavirus strain has been reported (*4*). However, little information on rotavirus groups E–G has been reported (*1**–**3**,**5**,**6*).

Human rotavirus strains J19 and B219, which are not classified into group A, B, or C, have been detected in China and Bangladesh (*7**–**11*). Strain J19, which was detected during a large epidemic of diarrhea in adults in China in 1997, has been propagated in human embryo kidney cells (*7**,**8*). Complete nucleotide sequences of all 11 RNA segments of strain J19 have been determined (*9*). In addition, the complete nucleotide sequence of the genome of Bangladesh strain B219 has been determined (*10**,**11*). Comparative sequence analysis showed that these 2 rotavirus strains are part of a novel group of rotaviruses.

We determined complete nucleotide sequences of the 4 RNA segments encoding viral protein 4 (VP4), VP6, VP7, and nonstructural protein 4 (NSP4) of a porcine rotavirus strain (SKA-1) from Japan (*12*) by using cDNA products obtained by a single-primer amplification method (*13**,**14*). Sequence data showed that SKA-1 is closely related to the novel group of human rotaviruses (J19 and B219).

## The Study

A fecal specimen was obtained from a piglet experimentally infected with strain SKA-1, which was first isolated from a pig with diarrhea in Tottori Prefecture, Japan. RNA was extracted from a 20% fecal suspension in phosphate-buffered saline by using the ISOGEN-LS Kit (Nippon Gene Ltd., Toyama, Japan). Cloning was performed according to the method described by Lambden et al. (*13*) with some modifications (*14*). During single-primer amplification, several DNA bands were detected that appeared to correspond to RNA segments of a rotavirus.

DNA was purified by using a Wizard SV Gel and a PCR Clean-Up System (Promega, Madison, WI, USA) and cloned into the PCR-TOPO vector by using a TOPO TA Cloning Kit (Invitrogen, Carslbad, CA, USA). Six clones for each of the segments were selected and used for sequencing. Sequence analysis and comparisons were performed by using GENETYX-WIN software (GENETYX, Tokyo, Japan) and MEGA software (www.megasoftware.net/).

We selected 4 genes encoding VP4, VP6, VP7, and NSP4 for sequence analysis. Sequence data were obtained for comparative sequence analysis among group A and nongroup A rotaviruses. There was little identity for any of the 4 genes between SKA-1 and group A or C rotaviruses. In contrast, nucleotide and amino acid sequences of the 4 genes of SKA-1 showed relatively high identities with those of a novel group of rotavirus strains (J19 and B219). The VP6 gene, which is associated with group specificity, of SKA-1 showed highest identities among the 4 genes with those of the novel group human rotaviruses: 72%–73% at the nucleotide level and 76%–77% at the amino acid level. VP6 also showed relatively high identities with those of group B rotaviruses: 52%–53% at the nucleotide level and 36%–40% at the amino acid level (Table). The other 3 genes (VP4, VP7, and NSP4), also showed high identities with those of the novel group of human rotaviruses, although identity values were lower than those for the VP6 gene (Table).

**Table:** Identities of nucleotide and amino acid sequences of VP4, VP6, VP7, and NSP4 of porcine rotavirus strain SKA-1 with those of group B and a novel group of rotaviruses*

Strain	Species	Group	% Identity of nucleotide (amino acid) sequences			
			VP4	VP6	VP7	NSP4
J19	Human	Novel	59.3 (52.0)	72.9 (76.8)	64.4 (56.3)	62.1 (36.1)
B219	Human	Novel	58.3 (51.7)	72.2 (76.5)	64.6 (55.9)	62.3 (35.2)
Bang 373	Human	B	48.6 (29.5)	52.5 (37.3)	48.4 (21.8)	48.4 (17.3)
WH-1	Human	B	48.8 (29.0)	52.7 (37.9)	48.9 (21.8)	49.3 (16.8)
ADRV	Human	B	49.1 (28.6)	52.7 (36.9)	49.1 (21.8)	49.4 (16.8)
RUBV226	Bovine	B	47.8 (29.8)	52.4 (39.2)	NA	NA
DB176	Bovine	B	47.3 (29.9)	52.4 (39.4)	50.7 (21.4)	NA
Nemuro	Bovine	B	NA	52.6 (38.2)	49.6 (20.2)	NA
Po/PB-F18	Porcine	B	NA	NA	(21.4)†	NA

*VP, viral protein; NS, nonstructural protein; NA, not available. GenBank accession nos.: SKA-1 VP4, AB576625; SKA-1 VP6, AB576626; SKA-1 VP7 AB576627; SKA-1 NSP4; AB576628. †Nucleotide sequence of the noncoding region of this strain was not available.

Lengths and nucleotide sequences of the 5′ and 3′ noncoding regions of the 4 genes of SKA-1 were similar to those of the novel group of rotaviruses. Using the VP7 gene as a reference, we identified sequences of the 5′ noncoding region of VP7 as 5′-GGAACTTTAAAGCC-3′ for strain SKA-1, 5′-GGCAATTTGAAGCC-3′ for the novel group of human rotaviruses, and 5′-GGCAATAAA-3′ for group B rotaviruses. Phylogenetic analysis of the 4 genes also showed that SKA-1 is closely related to the novel group of human rotavirus strains (J19 and B219) (Figure).

**Figure:**
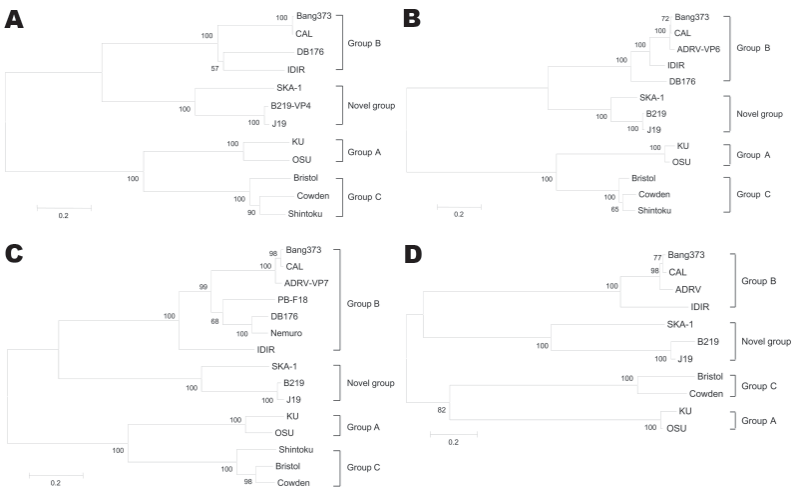
Phylogenetic trees for A) viral protein (VP) 4, B) VP6, C) VP7, and D) nonstructural protein 4 genes of group A, B, and C rotaviruses, a novel group of human rotaviruses, and porcine rotavirus strain SKA-1. Scale bars indicate nucleotide substitutions per site.

## Conclusions

Although group A rotaviruses have been extensively studied, nongroup A rotaviruses have not been extensively studied. In particular, there is little information on group D, E, F, and G rotaviruses. A novel group of human rotaviruses (J19 and B219) are not related to any other groups of rotaviruses.

Rotavirus strain SKA-1 was detected in a fecal specimen from a piglet with diarrhea in Japan, and was isolated in MA-104 cells in 1999. Its group specificity was suggested to be group B on the basis of reverse transcription PCR results with a group B–specific primer. However, our sequence analysis showed that the porcine SKA-1 strain is different from group B human and porcine rotaviruses.

Nucleotide and amino acid sequences of SKA-1 did not show identities with those of group A or C rotaviruses, although they showed some relatedness to those of group B rotaviruses. However, high identities were observed between SKA-1 and a novel group of human rotaviruses. In addition, there are similarities in the 4 genes analyzed in this study between SKA-1 and the novel group of human rotaviruses: 1) nucleotide sequences and nucleotide numbers of noncoding regions at the 5′ and 3′ ends are similar to each other; 2) lengths of nucleotide and deduced amino acid sequences are similar to each other; and 3) phylogenetic analysis showed that these viruses are in the same cluster.

Except for porcine strain SKA-1, there has been no report of animal rotaviruses being classified into a novel group of human rotaviruses. A survey of the prevalence of antibodies against SKA-1 among humans and animals, including pigs, would be useful. A classification system for nongroup A rotaviruses has not been established because 1) information on nucleotide sequences of group D, E, F, and G rotaviruses is lacking; 2) no expressed reference VP6 proteins are available; 3) reference strains of group D–G rotaviruses have not been adapted to cell culture; and 4) it is not known whether fecal samples or RNA or extracted RNA of group E–G strains are available in any laboratories. On the basis of serologic characterization and sequence analysis, a classification system for nongroup A rotaviruses, which includes the novel group of human and porcine rotaviruses such as J19, B219, and SKA-1, should be established.

## References

[1] Estes MK, Kapikian AZ. Rotaviruses. In: Knipe DM, Howley PM, editors. Fields Virology. 5th ed., vol 1. Philadelphia: Lippincott Williams & Wilkins; 2006. p. 1917–74.

[2] Saif LJ, Jiang B. Nongroup A rotaviruses of humans and animals. Curr Top Microbiol Immunol. 1994;185:339–71. 8050284 10.1007/978-3-642-78256-5_11

[3] Ramig RF, Ciarlet M, Mertens PPC, Dermody TS. Genus *Rotavirus*. In: Fauquet CM, Mayo MA, Maniloff J, Desselberger U, Ball LA, editors. Virus taxonomy. Eighth Report of the International Committee on Taxonomy of Viruses. New York: Elsevier Academic Press; 2005. p.484–96.

[4] Trojnar E, Otto P, Roth B, Reetz J, Johne R. The genome segments of group D rotavirus possess group A–like conserved termini but encode group-specific proteins. J Virol. 2010;84:10254–65. 10.1128/JVI.00332-10 20631147 PMC2937790

[5] Pedley S, Bridger JC, Brown JF, McCrae MA. Molecular characterization of rotaviruses with distinct group antigens. J Gen Virol. 1983;64:2093–101. 10.1099/0022-1317-64-10-2093 6311947

[6] Pedley S, Bridger JC, Chasey D, McCrae MA. Molecular definition of two new groups of atypical rotaviruses. J Gen Virol. 1986;67:131–7. 10.1099/0022-1317-67-1-131 3003232

[7] Yang H, Chen S, Ji S. A novel rotavirus causing large scale of adult diarrhea in Shi Jiazhuang [in Chinese]. Zhonghua Liu Xing Bing Xue Za Zhi. 1998;19:336–8. 10921117

[8] Ji S, Bi Y, Yang H, Yang F, Song J, Tao X, Cultivation and serial propagation of a new rotavirus causing adult diarrhea in primary human embryo kidney cells [in Chinese]. Zhonghua Yi Xue Za Zhi (Taipei). 2002;82:14–8. 11953119

[9] Jiang S, Ji S, Tang O, Cui X, Yang H, Kan B, Molecular characterization of a novel adult diarrhea rotavirus strain J19 isolated in China and its significance for the evolution and origin of group B rotaviruses. J Gen Virol. 2008;89:2622–9. 10.1099/vir.0.2008/001933-0 18796732

[10] Alam MM, Kobayashi N, Ishino M, Ahmed MS, Ahmed MU, Paul SK, Genetic analysis of an ADRV-N-like novel rotavirus strain B219 detected in a sporadic case of adult diarrhea in Bangladesh. Arch Virol. 2007;152:199–208. 10.1007/s00705-006-0831-y 16900303

[11] Nagashima S, Kobayashi N, Ishino M, Alam MM, Ahmed MU, Paul SK, Whole genomic characterization of a human rotavirus strain B219 belonging to a novel group of the genus *Rotavirus.* J Med Virol. 2008;80:2023–33. 10.1002/jmv.21286 18814255

[12] Sanekata T, Kuwamoto Y, Akamatsu S, Sakon N, Oseto M, Taniguchi K, Isolation of group B porcine rotavirus in cell culture. J Clin Microbiol. 1996;34:759–61. 8904456 10.1128/jcm.34.3.759-761.1996 PMC228888

[13] Lambden PR, Cooke SJ, Caul EO, Clarke IN. Cloning of noncultivatable human rotavirus by single primer amplification. J Virol. 1992;66:1817–22. 1371174 10.1128/jvi.66.3.1817-1822.1992 PMC240952

[14] Wakuda M, Pongsuwanna Y, Taniguchi K. Complete nucleotide sequences of two RNA segments of human picobirnavirus. J Virol Methods. 2005;126:165–9. 10.1016/j.jviromet.2005.02.010 15847933

